# Searching for Virulence Factors among *Staphylococcus lugdunensis* Isolates from Orthopedic Infections: Correlation of *β-hemolysin, hemolysin III*, and *slush* Genes with Hemolytic Activity and Synergistic Hemolytic Activity

**DOI:** 10.3390/ijms242115724

**Published:** 2023-10-29

**Authors:** Stefano Ravaioli, Davide Campoccia, Rasoul Mirzaei, Valentina Mariani, Giulia Bottau, Andrea De Donno, Lucio Montanaro, Pietro Speziale, Carla Renata Arciola

**Affiliations:** 1 Laboratorio di Patologia delle Infezioni Associate all’Impianto, IRCCS Istituto Ortopedico Rizzoli, Via di Barbiano 1/10, 40136 Bologna, Italy; davide.campoccia@ior.it (D.C.); valentina.mariani@ior.it (V.M.); giulia.bottau@ior.it (G.B.); andrea.dedonno@ior.it (A.D.D.); lucio.montanaro@unibo.it (L.M.); 2 Venom and Biotherapeutics Molecules Laboratory, Medical Biotechnology Department, Biotechnology Research Center, Pasteur Institute of Iran, Tehran 1316943551, Iran; rasul.micro92@gmail.com; 3 Laboratory of Immunorheumatology and Tissue Regeneration, Laboratory of Pathology of Implant Infections, IRCCS Istituto Ortopedico Rizzoli, Via di Barbiano 1/10, 40136 Bologna, Italy; 4 Department of Molecular Medicine, Biochemistry Section, Viale Taramelli 3/b, 27100 Pavia, Italy; pietro.speziale@unipv.it; 5 Department of Medical and Surgical Sciences (DIMEC), University of Bologna, Via San Giacomo 14, 40126 Bologna, Italy

**Keywords:** *Staphylococcus lugdunensis*, *slush* genes, hemolysis, synergistic hemolytic activity, β-hemolysin, δ-hemolysin, virulence factors, real time PCR, sequencing

## Abstract

*Staphylococcus lugdunensis* is an emerging high-virulent pathogen. Here, the presence and expression of virulence genes (*icaA*, *fbl*, *vwbl*, *fbpA*, *slush A*, *B* and *C*, and genes of the putative *β-hemolysin* and *hemolysin III*) and the ability to induce synergistic hemolytic activity and hemolysis after 24, 48 and 72 h were investigated in a collection of twenty-two *S. lugdunensis* clinical isolates. The collection of isolates, mainly from implant orthopedic infections, had previously been grouped by ribotyping/dendrogram analysis and studied for biofilm matrices, biomasses and antibiotic resistances. Two isolates, constituting a unique small ribogroup sharing the same cluster, exhibited an amplicon size of the *slush* operon (*S. lugdunensis* synergistic hemolysin) which was shorter than the expected 977 bp. This outcome can predict the genetic lineage of the *S. lugdunensis* strains. One isolate (*cra*1342) presented two deletions: one of 90 bp in *slush A* and the other of 91 bp in *slush B*. Another isolate (N860314) showed a single 193 bp deletion, which encompassed part of the *slush B* terminal sequence and most of *slush C*. The isolate N860314 was devoid of hemolytic activity after 24 h, and the first consideration was that the deleted region deals with the coding of the active enzymatic site of the slush hemolysin. On the other hand, *cra*1342 and N860314 isolates with different *slush* deletions and with hemolytic activity after 24 and 48 h, respectively, could have replaced the hemolytic phenotype through other processes.

## 1. Introduction

*Staphylococcus lugdunensis* is a coagulase-negative *Staphylococcus* (CoNS), which is increasingly reported as a prominent human opportunistic pathogen. Its name derives from “Lugdunum”, the Latin name of Lyon, the French city where in 1988 a clinical strain of *Staphylococcus lugdunensis* was isolated for the first time [1].

*S. lugdunensis* has emerged as the most virulent CoNS species exhibiting pathological and clinical features that make it closer to *Staphylococcus aureus* than to other CoNS species. *S. lugdunensis* shares more than 70% of its genome with *S. aureus* [2]. *S. lugdunensis* has been represented as a “wolf in sheep’s clothing” as it causes many serious diseases, such as skin and soft tissue infections (SSTI), subcutaneous tissue infections, bone and joint infections, prosthetic joint infections (PJI), vascular catheter-related infections, infective endocarditis (IE), bacteremia and abscesses [3,4,5,6,7,8,9,10]. Although *S. lugdunensis* is susceptible to most antibiotics [11,12,13,14] and lacks many of the virulence factors that *S. aureus* possesses, such as protein A, enterotoxins A, B or C, toxic shock syndrome toxin (TSST), hemagglutinin and toxin [1,15], this wolf in disguise appears to be equipped with other virulence factors and is capable of expressing pathological mechanisms. *S. lugdunensis* can adhere to host matrix proteins with surface adhesins such as the fibrinogen-binding surface protein (Fbl) [16], the putative fibronectin/fibrinogen binding proteins (fbpA), the von Willebrand factor binding protein (vWbf) [17], and the polysaccharide intercellular adhesin (ica) [18] and can produce some cytolytic toxins toward human erythrocytes such as the putative β-hemolysin [19], the putative hemolysin III [20,21], and the small cytolytic *S. lugdunensis* synergistic hemolysins (SLUSH), peptides with δ-toxin-like activity [9,17]. The activity of *S. lugdunensis* δ-hemolysin derives from the three peptides SLUSH-A, -B and -C encoded by three linked genes within the *slush* locus, which is distinct to the δ-hemolysin of *S. aureus* with homologies with three peptides produced by *Staphylococcus hemolyticus* [9,21,22]. SLUSH peptides belong to the group of phenol-soluble modulins (PSMs) produced by *S. aureus*. They can act synergistically to enhance the lysis of erythrocytes with the *S. aureus* β-toxin (sphingomyelinase C) and probably with the orthologue of *S. lugdunensis* β-toxin [9]. Hébert G.A in 1990 disclosed for the first time that δ-hemolysin of *S. lugdunensis* potentiated the zone of a β-hemolysin of *Staphylococcus intermedius* producing a complete hemolysis on agar containing sheep erythrocytes [22].

The aim of this study was to investigate: (1) the ability to induce synergistic hemolytic activity and hemolysis after 24, 48 and 72 h in a collection of 22 *S. lugdunensis* clinical isolates; (2) the relative expression of the virulence genes (*icaA*, *fbl*, *vwbl*, fbpA, *slush A*, *B* and *C*, and of the genes of the putative *β-hemolysin* and *hemolysin III*) in relation to the clonal lineages and to the hemolytic activity and the synergistic hemolytic activity and outcomes, and (3) the sequence of the shorter amplicon of the *slush* operon gene of two isolates belonging to the *cra*-129-S-8 ribogroup and its relation to the hemolytic activity and synergistic hemolytic activity and outcomes. The collection of isolates had previously been grouped by ribotyping/dendrogram analysis and studied for biofilm matrices, biomasses and antibiotic resistances.

## 2. Results

### 2.1. PCR

All the isolates were positive for all the genes tested, i.e., *fbl*, *vwbl*, *fbpA*, *icaA*, and for the putative *beta-hemolysin* and the putative *hemolysin III*.

### 2.2. PCR and Sequencing of Slush Gene

**Figure 1:**
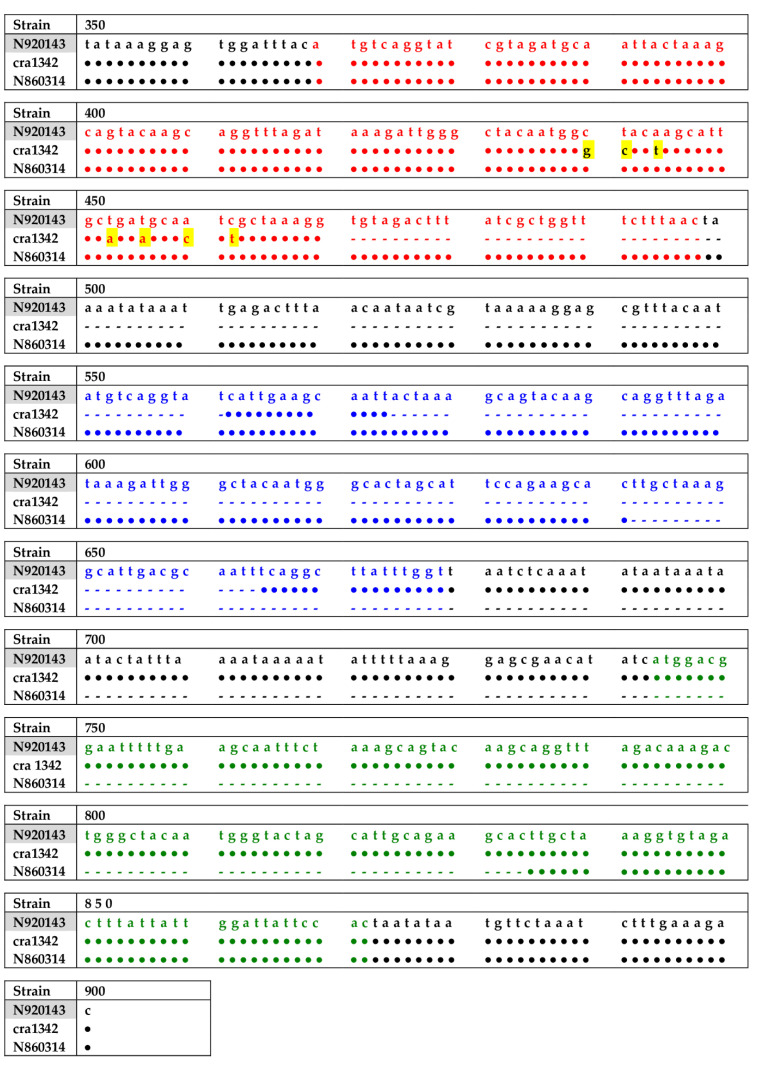
Gene sequence in the 350–900 bp region of the *slush* operon for the reference *S. lugdunensis* strain N920143 and the two clinical isolates *cra*1342 and N860314. In red characters the *slushA* gene; in blue characters the *slushB* gene; in green characters the *slushC* gene; **∙**, base matching with the reference gene sequence; -, deleted base; and base changes are highlighted in yellow.

PCR screening for the *slush* operon (synergic hemolysin) was positive for all strains except for the strain E319. Thus, further investigations are warranted before concluding that this isolate is defective for the entire operon and could be indicated as Δ*slush*. Moreover, the PCR analysis of the *slush* operon showed that two isolates, i.e., *cra*1342 and N860314, both belonging to cluster C and to the *cra*-129-S-8 ribogroup, exhibited an altered amplicon, shorter than expected. The analysis of the fragments’ sequence of the two isolates with the variant form of the *slush* operon gene showed deletions, compared to the sequence of *S. lugdunensis* N920143 used as a reference strain (Figure 1), of 181 bp (91 bp + 90 bp) and 193 bp, respectively. In particular, the sequence of *cra*1342 isolate had two deletions, one of 91 bp from the end of *slushA* gene to the beginning of *slushB* gene and the other of 90 bp internal to *slushB* gene, whereas the sequence of N860314 isolate had only one deletion of 193 bp involving the second half trait of *slushB* gene to the first half trait of *slushC* gene (Figure 1).

### 2.3. Quantitative Real-Time PCR (qPCR) of icaA, fbl, vwbl, fbpA, β-Hemolysin and Hemolysin III and (qPCR) of Slush A, B and C Genes

**Figure 2:**
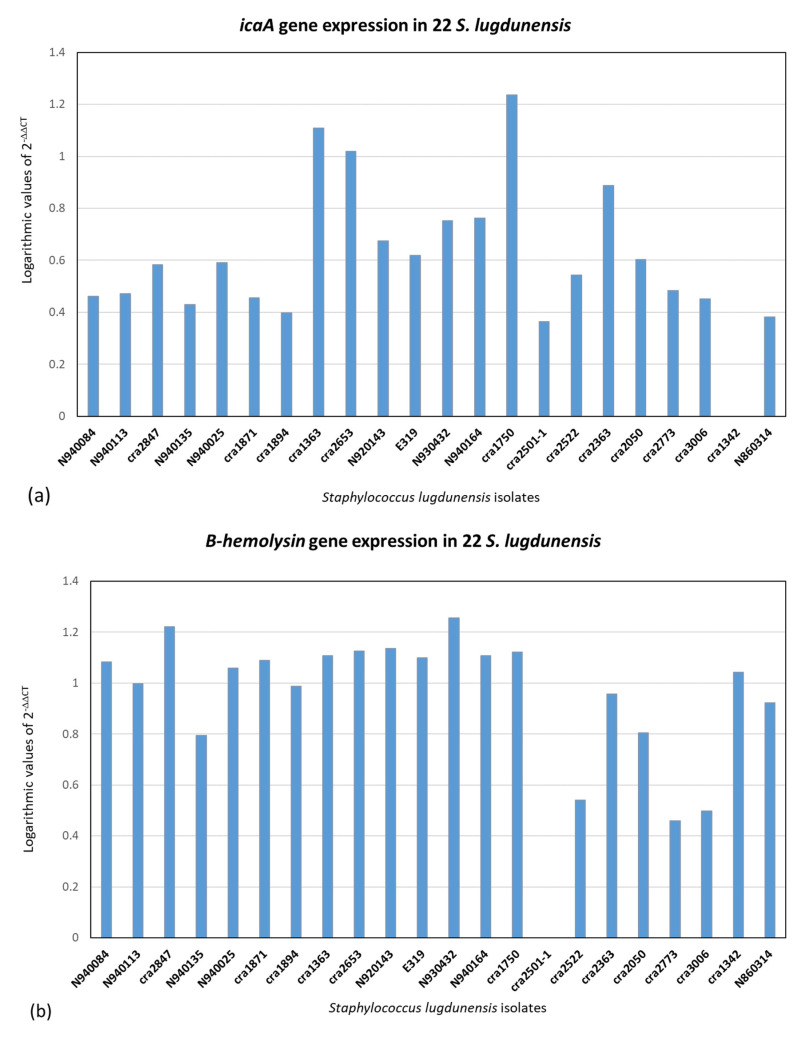
Relative gene expression using the 2^−ΔΔCT^ method with logarithmic values in 22 *S. lugdunensis* isolates. The internal target gene was *gmk*. The reference samples were chosen between those with lower gene expression. (**a**) *icaA* (ref. sample: *cra*1342). (**b**) *β-hemolysin* (ref. sample: *cra*2501).

**Figure 3:**
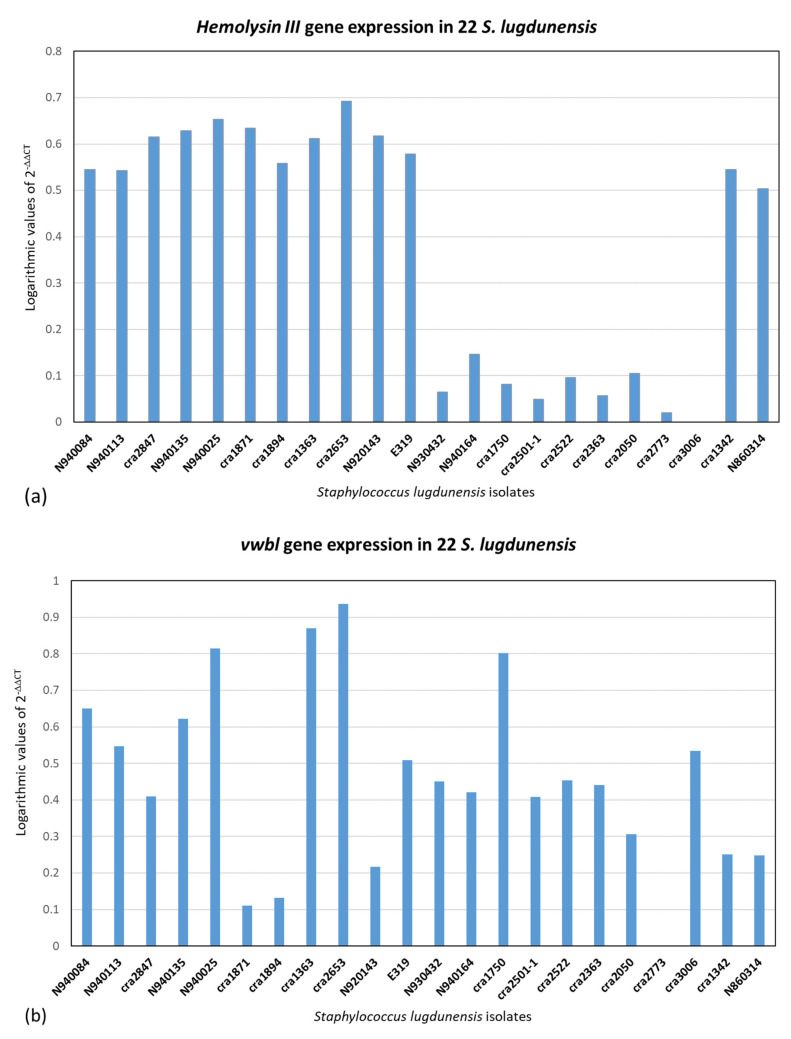
Relative gene expression using the 2^−ΔΔCT^ method with logarithmic values in 22 *S. lugdunensis* isolates. The internal target gene was *gmk*. The reference samples were chosen between those with lower gene expression. (**a**) *hemolysin III* (ref. sample: *cra*3006). (**b**) *vwbl* (ref. sample: *cra*2773).

**Figure 4:**
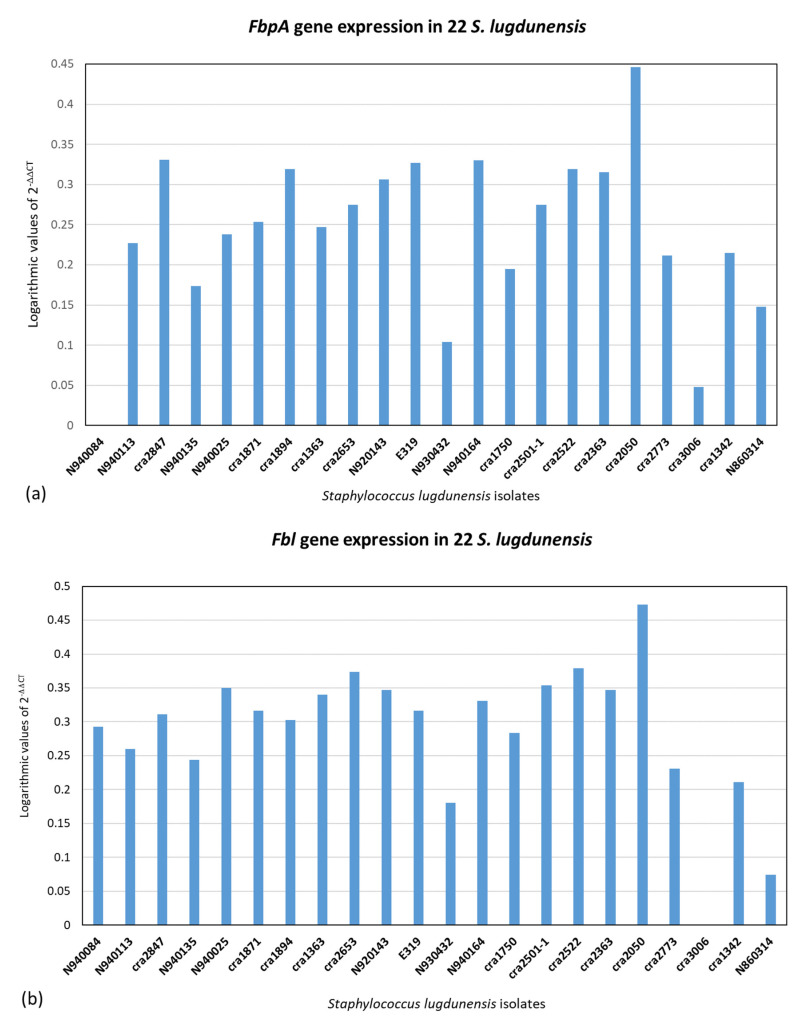
Relative gene expression using the 2^−ΔΔCT^ method with logarithmic values in 22 *S. lugdunensis* isolates. The internal target gene was *gmk*. The reference samples were chosen between those with lower gene expression. (**a**) *fbpA* (ref. sample: N2940084). (**b**) *fbl* (ref. sample: *cra*3006).

qPCR was executed on the 22 *S. lugdunensis* isolates. The 2^−ΔΔCT^ method was used for qPCR data analysis for relative gene expression. The results of 2^−ΔΔCT^ were represented in logarithmic scale. Figure 2, Figure 3 and Figure 4 illustrate the logarithmic 2^−ΔΔCT^ values of relative expression of each gene for all the *S. lugdunensis* isolates using samples that providing the lowest gene expression as a reference.

**Figure 5:**
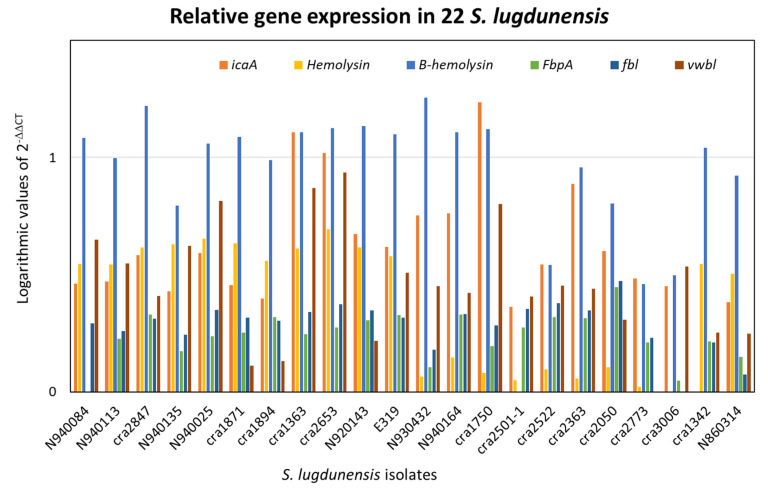
Distribution of relative gene expression with logarithmic values of *icaA*, *fbl*, *vwbl*, *fbpA*, *β*-*hemolysin* and *hemolysin III* genes in the 22 *S. lugdunensis* isolates studied. The 2^−ΔΔCT^ method was used, and *gmk* was the internal target gene.

Figure 5 indicates the distribution of the logarithmic 2^−ΔΔCT^ values for all the genes together with all the *S. lugdunensis* isolates. The acquired data do not reveal a distinct panel of gene expression related to the clonal evolution, i.e., ribotypes or clones found with dendrogram analysis, or to the clinical source or the territorial origin. Furthermore, there is not any correlation between the genes belonging to an isolate. Figure 2, Figure 3, Figure 4 and Figure 5 show dissimilar distribution of gene expression levels in the 22 *S. lugdunensis* between isolates of the same clone, the patterns are different and specific for each isolate. Some isolates have higher gene expression values for all the tested genes, such as *cra*2847 and *cra*2653, while others, such as *cra*3006, *cra*2773 and *cra*2501, have in general low gene expression. The isolate *cra*3006 exhibits the lowest *hemolysin III* and *fbl* gene expression and was used as the reference sample for those genes. N2940084 has the lowest *fbpA* gene expression but has a good amount of expression for the other genes tested. The isolate *cra*1750 shows a high amount of *icaA* and *β*-*hemolysin* gene expression but low *fbpA* and *hemolysin III* gene expression. The reference samples *cra*1342, *cra*2501 and *cra*2773 were applied for the *icaA*, *β*-*hemolysin* and *vwbl* genes, respectively.

**Figure 6:**
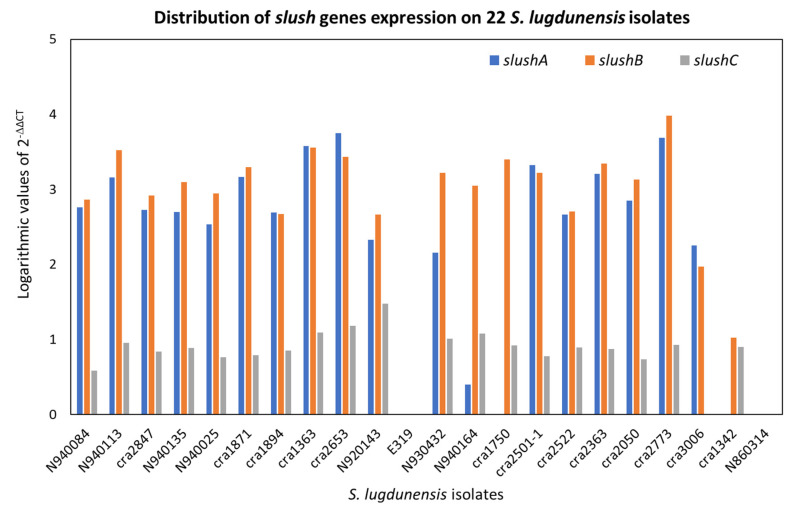
Distribution of *slush A*, *B* and *C* gene expression using the 2^−ΔΔCT^ method with logarithmic values in the 22 *S. lugdunensis* isolates studied. The internal reference gene used for qPCR was the *gmk* gene. The reference samples were chosen between those with the lower gene expression; they were E319 for *slush A* and *B* and *cra*3006 for *slushC*.

Figure 6 exposes the relative gene expression for *slushA*, *B* and *C.* In this analysis, the distribution of the amounts of gene expression is not related with some specific pattern, for example, belonging to a specific clone or to the hemolytic and synergy phenotype. In fact, all the *S. lugdunensis* isolates have a casual panel of *slush* genes.

As expected, the E319 isolate, defective for the entire *slush* operon, and the N860314 isolate, with a 193 bp deletion, do not express the *slush A*, *B* and *C* genes. Surprisingly, *cra*1342, with two deletions, one from the end of the *slushA* gene to the beginning of the *slushB* gene and the other internal to the *slushB* gene, does not express *slush A*, as expected, but does express *slush B*.

### 2.4. Hemolytic Test and Synergistic Activity

The test performed in duplicate exhibited the same results. The ability to induce hemolytic activity on Columbia blood agar plates after 24 h, 48 h and 72 h of incubation was investigated on the 22 *S. lugdunensis* isolates (Table 1).

At 24 h of incubation, the hemolysis test was positive for nine isolates, and negative for thirteen isolates, while at 48 h of incubation, the hemolysis test was positive for seventeen isolates and negative for five isolates. Therefore, following 24 h of incubation, another seven isolates with a negative hemolytic phenotype turned out to be positive. All the remaining five isolates with a negative hemolytic phenotype after 48 h of incubation had a slight positive activity after 72 h of incubation.

**Figure 7:**
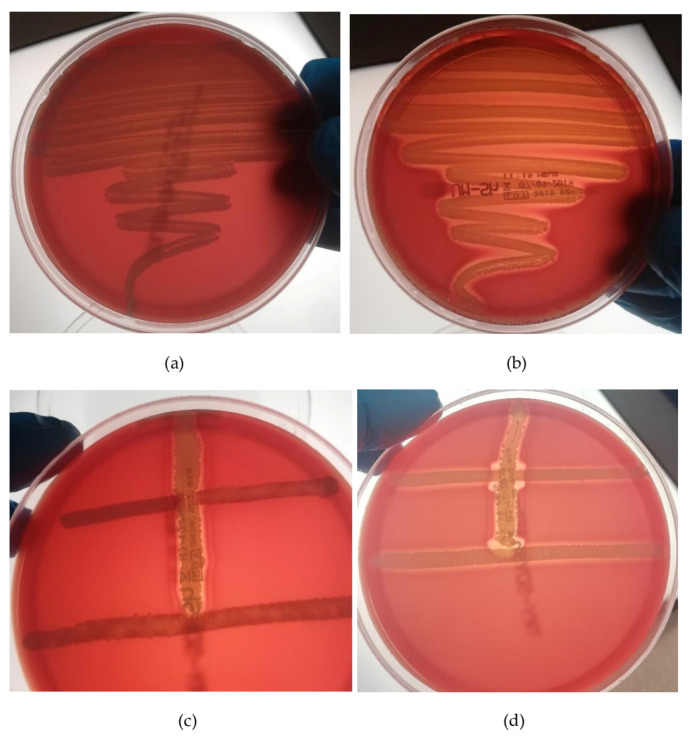
Negative (*S. lugdunensis* N860314) and positive (*S. lugdunensis cra*1342) hemolysis after 24 h (**a**,**b**). Absence of the synergistic activity between *S. lugdunensis* N930432 and *S. aureus* ATCC25923 strain (**c**) and complete synergistic hemolysis between *S. lugdunensis* N940025 and *S. aureus* ATCC25923 strain (**d**).

The isolates *cra*1342 and N860314, with the *slush* operon variants, were, respectively, positive and negative to the hemolysis test after 24 h and both were positive after 48 h (Figure 7). E319 is missing for the *slush* gene, and it is negative in the hemolysis test.

The ability to induce hemolytic activity due to the synergistic effect between the 22 *S. lugdunensis* isolates and the *S. aureus* ATCC25923 β-lysin producer was detected after 24 h.

*S. aureus* and *S. lugdunensis* create a wide zone of incomplete hemolysis all along their line of growth and a large zone of synergistic, complete hemolysis next to the intersection of the lines of *S. lugdunensis* and *S. aureus* growth due to the β-lysin and δ-lysin production in *S. lugdunensis*. Table 1 shows the relationship between the phenotypic results and the logarithmic values of the *slush* genes expression on the 22 *S. lugdunensis* isolates tested. No associations between the data were found and the synergistic hemolysis phenotype does not depend on the expression of *slushA*, *B* and *C* genes.

Synergistic hemolytic activity was detected on seventeen isolates, and negative activity was detected in five isolates. The unexpected combination of positive synergistic hemolytic activity with negative hemolytic phenotype after 24 h of incubation was observed in five isolates, whereas negative synergistic hemolytic activity was never detected with the positive hemolytic phenotype.

Four out of five strains, namely N920143, E319, N930432 and N940164, which exhibited a phenotype of negative synergistic hemolysis, also showed a negative hemolytic phenotype after 72 h.

Furthermore, of the three isolates defective for the *slush* operon, only the E319 strain showed a negative synergistic hemolytic phenotype; on the contrary, the N860314 isolate, with a positive synergistic hemolytic phenotype, did not express the *slushA*, *B* or *C* genes.

## 3. Discussion

The ability to induce synergistic hemolytic activity and hemolysis after 24, 48 and 72 h was analysed across all the *Staphylococcus lugdunensis* isolates. Four out of 22 isolates (N920143, E319, N930432 and N940164) that were positive for a *slush* operon showed neither synergistic hemolytic activity nor hemolytic activity after 48 h, while *cra*3006 showed no hemolytic activity after 48 h and *cra*2773 was unable to induce the synergistic hemolytic activity (Table 1). The synergistic hemolytic activity was not related to the ability to produce different biofilm matrix components, which was studied in our previous work [23]. These results were unexpected as *S. lugdunensis* synergistic hemolysins are peptides that cause red blood cell lysis [21]. It is probable that a lack of expression or genomic mutation, even when undetected by PCR analysis, may have caused these ineffective phenotypes.

The two isolates *cra*2773 and N860314 belonging to *cra*129-S8 ribogroup and to the cluster C, in accordance with our previous study [23], revealed an altered size of the *slush* operon gene suggesting that it could be accompanied to clonal evolution. The deletions in the *slush* operon are different from those previously observed by Didi J. et al. [24], who reported a 125 bp deletion between *slushB* and *slushC* genes. In this study, the *cra*1342 isolate shows two deletions, one of 91 bp between the end of the *slush A* and the beginning of the *slushB* gene and another of 90 bp internal to the *slushB* gene, while the N860314 isolate exhibits a unique large deletion of 193 bp regarding a region from the middle of the *slushB* to the *slushC* gene (Figure 1). This new finding is fascinating in terms of molecular epidemiology studies since it can predict the genetic lineage of the *S. lugdunensis* strains. In the literature, other studies established that *S. lugdunensis* genetic lineages are strictly associated with the prevalence of specific genetic traits. Lin L.C. et al. [25] demonstrated that the copy number of *vWbl* gene repeats in *S. lugdunensis* are associated with the same strain molecular types assessed by multi-locus sequence typing (MLST) and staphylococcal chromosome cassette *mec* (SCC*mec*)-typing and may be correlated with the pathogenicity. Another study showed that strains belonging to SCC*mec* II, and sequence type 6 (ST6) genotypes had unique mobile genetic elements (MGEs) encoding for a putative virulence factor and antimicrobial resistance genes [26]. This attractive characteristic needs to be confirmed; therefore, the subsequent strains that will be revealed to belong to the *cra*129-S8 ribogroup will be investigated for the presence of the altered size of the *slush* operon gene.

Furthermore, *cra*1342 and N860314 isolates, despite having a variant of the *slush* operon, showed a hemolytic phenotype after 24 and 48 h, respectively, and both isolates exhibited synergistic hemolytic activity. As a first interpretation, since the isolate N860314 was devoid of hemolytic activity after 24 h, it was suggested that the deleted region deals with the coding of the active enzymatic site of the *slush* hemolysin. However, this outcome is not surprising since other strains with incomplete *slush* operons showing a hemolytic phenotype have previously been described [17,24]. The prevalence of genes and their expression does not definitely predict the expected phenotype, which is probably because of the epigenetic regulations, environmental conditions and bacterial signals. As previously seen, the insertion/excision of genetic elements or short DNA sequences is not always the natural mechanism and the reason for the off/on switching of a genetic locus or operon and then the observed phenotype [27]. Our finding suggests either that the deleted sequence of *slush* operon is not directly implicated in hemolysis or else other compensatory genes may be involved in the hemolytic activity [24,28]. The reason why N860314 displayed a hemolytic phenotype after 48 h while *cra*1342 displayed a hemolytic phenotype after 24 h is not connected with the altered size of the *slush* operon gene and should be explained through other mechanisms. Other isolates that were positive for the *slush* operon gene showed a hemolytic phenotype after 48 h, whereas the ones without a hemolytic phenotype after 48 h displayed only weak hemolysis after 72 h (Table 1).

The reason why *cra*1342 and N860314 isolates with incomplete *slush* operon (Δ*slushA*, Δ*slushB*, and *slushC* and *slushA*, Δ*slushB*, and Δ*slushC,* respectively) showed the synergistic hemolytic activity with *S. aureus* could be explained by the preservation of one *slush* gene, which may be adequate to provide this synergy.

Real time PCR analysis for *slushA*, *B* and *C* gene products was executed with the aim of investigating if the isolates that were negative on the hemolysis assay and synergistic hemolytic activity had a gene expression deficit. N860314 did not show any gene expression for *slushA*, *B* or *C* genes, while *cra*1342 did not show gene expression only for the *slush*A gene, but both exhibited hemolytic activity (Figure 6). On the contrary, three out of the five isolates that had a pronounced gene expression for all the *slush* (*slushA*, *B* and *C*) genes were negative for hemolytic activity; therefore, the outcomes pointed out that some other mechanism is involved in hemolysis activity and should be defined. Chin D. et al. [29] established that accessory gene regulator A (*agrA*) is the major regulator of hemolysins in *S. lugdunensis*, being the positive regulator of SLUSH peptides, and that Δ*agrA*, but not the *slush* gene deletion, enhances the susceptibility to killing by whole human blood. The *hemolysin III* and *β-hemolysin* gene expression, in addition to the *slush A*, *B* and *C* gene expression results, were found to be insufficient in elucidating the reason of the different hemolytic phenotypes of *S. lugdunensis* isolates.

Real-time PCR was used on the tested virulence genes to look for a relationship between the gene expression values resulting from this technique and the genetic lineages created by riboprinting (Figure 2, Figure 3, Figure 4 and Figure 5). The clonal population structure of the *S. lugdunensis* strains is not related to the gene expression of virulence traits except for the *hemolysin III*, where a connection between the strains belonging to the *cra*62-S1 and *cra*129-S8 ribogroups and a high gene expression is evident. The strains belonging to each ribogroup do not have a definite panel of virulence factors, as already seen in our previous study where *S. lugdunensis* ribogroups were not related with the various biofilm matrix components [23]. Ortega-Peña S. et al. (2019) and Sanchez A. et al. (2020) found that in *S. epidermidis* there are genetic traits that could serve as biomarkers to differentiate clinical from commensal isolates [30,31]. Other authors argued that genotyping fails to identify hypervirulent and invasive genetic lineages or clusters [18,24,32]. This contrasts with our previous findings in *S. aureus* and *S. epidermidis*, where it was possible to correlate clonal complexes with pathogenic characteristics and antibiotic resistances [33,34,35]. Similar gene expression of virulence factors in genetically related bacterial strains is associated with a greater competence to promote the survival of the bacterial clone and thus to be more relevant from an epidemiological point of view than the expression of other virulence factors. We can speculate that the expression of virulence factors that are not significant for the survival and affirmation of bacterial strains belonging to the same bacterial clone may be present randomly.

In this connection, it should also be considered that studying the molecular epidemiology of emerging pathogens, such as *S. lugdunensis*, favors the search for new anti-infective molecules alternative to antibiotics with which to coat or load biomaterials to make them capable of preventing and combating implant infections [36,37,38,39]. And indeed, grasping the clonal complexity promotes the transition to precision medicine, which adapts anti-infective biomaterials to the peculiar pathogenetic context [33,34,35,40,41,42].

Much remains to be elucidated on the actual role played by *S. lugdunensis* synergistic hemolysins as virulence determinants in humans and animals. *S. lugdunensis* strains have been proven to be hemolytic toward human erythrocytes but not toward murine erythrocytes [43]. There is converging evidence that *agrA* is a strong positive regulator of the expression of slush hemolysins [29,44]. The lack of hemolytic activity on murine erythrocytes would suggest overt human tropism for *S. lugdunensis*. The activity of *S. lugdunensis* synergistic hemolysins on other human cells such as leukocytes remains poorly investigated. Nonetheless, a series of interesting in vitro experiments conducted by Chin et al. [29] demonstrated that the deletion of the *slush* locus does not alter *S. lugdunensis* resistance to killing by whole human blood and by RAW 264.7 murine macrophages. Conversely, the deletion of *agrA* determined a reduction in the fitness of *S. lugdunensis* when exposed to RAW 264.7 and primary human M-CSF-derived macrophage but not when exposed to primary M1 polarized human macrophages and primary human neutrophils. Therefore, *S. lugdunensis* resistance to killing in the presence of phagocytes seems associated with other virulence factors controlled by agrA rather than with slush expression.

## 4. Materials and Methods

### 4.1. Species Identification and Storage

The investigation was executed on the 22 *S. lugdunensis* isolates described in our previous work [23]. Briefly, thirteen of the isolates (*cra* series) were collected at the Research Unit on Implant Infections of IOR (Bologna, Italy). Eight isolates (N series) were donated to one of the authors by Prof. François Vandenesch from the Centre National des Staphylocoques (Lyon, France). The E319 strain was provided by the Unit of Biochemistry (University of Pavia).

### 4.2. Staphylococcus Lugdunensis Subtyping and Biofilm Characterization

All 22 *S. lugdunensis* isolates were analysed and subtyped by automated ribotyping using a RiboPrinter^®^ Microbial Characterization System (Qualicon, Wilmington, DE, USA) and the patterns obtained were imported and analysed in BioNumerics version 7.0 (Applied Maths, Sint-Martens-Latem, Belgium) as executed in the earlier study of Ravaioli S. et al. [23]. The isolates were processed to detect the biomass and the eDNA, protein and exopolysaccharide biofilm’s components, as earlier performed in Ravaioli S. et al. [23]. In addition, information on the clinical diagnosis and antibiotic resistance panel is available from the same study.

### 4.3. Bacterial DNA Isolation

The chromosomal DNA used as an amplification template was extracted from the bacterial cultures using QIAmp DNA mini kit (Qiagen, GmbH, Hilden, Germany), according to the manufacturer’s instructions.

### 4.4. PCR

The isolates were screened and characterized for a panel of adhesin and hemolysin genes, including *fbl*, encoding the fibrinogen binding protein; *vwbl*, encoding the von Willebrand factor binding protein precursor; *fbpA*, encoding the putative fibronectin/fibrinogen binding protein; the putative *β-hemolysin* gene; and the putative *hemolysin III* gene and the *slush* operon, encoding the synergistic hemolysin. The primers used and the PCR amplifications were as reported by [17]. Amplified products were analysed on 1.5% agarose gels. Additionally, the isolates were screened for the presence of PIA by PCR amplification of a 909 bp fragment of the *icaA* gene (part of the *ica*-locus associated with the production of PIA exopolysaccharide) using the following primers: 5′-GGGAGCTCTGACAATTCTGC-3′ (forward) and 5′-GGCAGAAATAGCGACCAAAG-3′ (reverse). The primers used were created by the online primer designing tool “Primer3” “http://bioinfo.ut.ee/primer3-0.4.0/” (accessed on November 2011) using the sequence of *icaA* gene of *S. lugdunensis* HKU09-01 strain.

The primer sequences were confirmed by “BLAST” (Basic Local Alignment Sequence Tool), “http://blast.ncbi.nlm.nih.gov/Blast.cgi?PROGRAM=blastn&PAGE_TYPE=BlastSearch&LINK_LOC=blasthome” (accessed on November 2011). The amplification was optimized and carried out with a 2 min heating step at 95 °C, followed by 25 cycles of 60 s at 95 °C for denaturation, 60 s at 57 °C for primer annealing, 60 s at 72 °C for extension, and then 5 min at 72 °C for final extension. Amplified product was analysed on 1.5% agarose gels.

### 4.5. Slush Gene Amplicons Sequencing

Amplicons from the PCR reaction of the *slush* gene of the *cra*1342 and N860314 isolates were sent to be purified and processed in both directions by Eurofins MWG Operon (Ebensburg, Germany). The sequence of the *slush* operon gene of the isolate *S. lugdunensis* N920143 of the collection that regularly exhibited the expected 977 bp amplicon size was adopted as a positive control. The entire sequence of the *slush* gene of N920143 strain obtained from PubMed website database (accession number FR870271, EMBL database) was used as the reference for comparison with the two amplicons that lacked the DNA sequences. Unipro UGENE software (http://ugene.net/ (accessed on November 2011)) was used for the alignments and for detecting the missing sequences.

### 4.6. Quantitative Real-Time PCR of icaA, fbl, vwbl, fbpA, β-Hemolysin and Hemolysin III

The chromosomal RNA was extracted, for the analysis and quantification of gene expression, from bacterial cultures using GeneJET RNA Purification Kit (ThermoFisher Scientific, Life Technologies, Paisley, UK), according to the manufacturer’s instructions. For RNA isolation, bacteria cells were harvested after 4 h of cultures refreshed from overnight cultures, during the exponential phase of growth (OD_600_ = 0.5–1). RNA extracts of the 22 *S. lugdunensis* isolates were sent to be processed for the Real Time quantitative PCR (qPCR) by Open Lab s.r.l. (Bologna, Italy), which designed the TaqMan Custom assays taking all the gene sequences as references. SuperScript™ VILO™ Master Mix (ThermoFisher Sc., Life Technologies, Carlsbad, CA, USA) was used for the reverse transcription and TaqMan™ Fast Advanced Master Mix for the qPCR. The internal reference gene used for qPCR was the *gmk* gene (guanylate kinase), a housekeeping gene of MLST (Multi Locus Sequence Typing) analysis. The analysis was performed in duplicate using the QuantStudio™ 3 Real-Time PCR System (ThermoFisher Sc., Life Technologies, Singapore). The 2^−ΔΔCT^ method has been used as the relative quantification strategy for qPCR data analysis [45]. ΔCT is the difference in threshold cycle averages between the target and reference genes (1).

ΔCT = CT (a target gene) − CT (a reference gene)(1)

The ΔΔCT is the difference in ΔCT as described in the above formula between the target and reference samples (2). It was used as reference samples in the ones showing the lower gene expression for the analysed gene.

ΔΔCT = ΔCT (a target sample) − ΔCT (a reference sample)(2)

The result of this method is presented as the fold change in the target gene expression in a target sample in relation to the chosen reference sample. The relative gene expression for reference samples is set to 1 because ΔΔCT is equal to 0 (2^0^ = 1). The target samples with CT after 31 cycles have been considered without any gene expression, following the recommendation of the company consultant.

### 4.7. Quantitative Real-Time PCR (qPCR) of Slush A, B and C Genes

#### 4.7.1. Primers and Probe Design

The primers and probe for *slush* A, B and C genes were designed by the consultant of “IDT- TEMA Ricerca” (TEMA Ricerca, Castenaso, Bologna, Italy) (received the 21 January 2021) (Table 2). Unique primers and probes were designed for each of the three targets. Affinity Plus™ qPCR Probes for enhanced discrimination of thermodynamically similar samples such as single nucleotide polymorphisms and transcript variants was used. The probes included Affinity Plus bases (indicated by the plus signs) to enhance specificity between the three targets. The 5’-reporter dye was 6-FAM™ and the 3’-quencher was Iowa Black^®^ FQ. The 1.5 µL of primer mix used for the qPCR reaction was constituted by 5 µL fw + 0.5 µL rev + 0.5 µL probe.

#### 4.7.2. Quantitative Real-Time PCR Conditions

Reverse transcription of RNA into cDNA was performed with an iSCRIPT gDNA clear cDNA Synthesis kit (Bio-Rad Laboratories s.r.l., Segrate, Milano, Italy). Quantitative real-time PCR was performed in duplicate using the QuantStudio™ 5 Real-Time PCR System (ThermoFisher Sc., Life Technologies, Singapore). Each reaction tube contained 20 µL of reaction mixture, including 10 μL of 2× TaqMan Fast Advanced Master Mix (ThermoFisher Sc., Life Technologies, Paisley, UK), 1.5 µL of 10 µM primer mix (0.25 µM as final concentration) and 8.5 µL of cDNA with nuclease-free water to have 500 ng of cDNA. The internal reference gene used for qPCR was the *gmk* gene (guanylate kinase). The QuantStudio™ 5 machine was programmed as follows: UNG incubation at 50 °C for 2 min, a polymerase activation at 95 °C for 2 min, followed by 40 cycles of 20 s of denaturation at 95 °C and 60 s annealing/extension at 60 °C.

### 4.8. Hemolysis Test

A colony from the 22 *S. lugdunensis* isolates formerly seeded in Tryptic Soy Agar (Meus, Vacutest Kima s.r.l., Arzergrande, Padova, Italy) were soaked into Tryptone Broth (Biolife Italiana s.r.l., Mascia Brunelli spa, Milano, Italy) and incubated overnight at 37 °C. To investigate the hemolytic activity, a loop of the bacterial suspension was plated onto Columbia blood agar plates containing 5% sheep blood (BioMérieux Italia, Bagno a Ripoli, Firenze, Italy) and grown for 24 and 48 h of incubation at 37 °C. The test was performed in duplicate.

### 4.9. Synergistic Hemolysis Growth Test

To investigate the synergistic hemolytic activity of the delta-like hemolysin of *S. lugdunensis* isolates, the *S. aureus* ATCC25923 strain producing beta-hemolysin was used. Briefly, a loop of the bacterial suspension of *S. aureus* was streaked on a Tryptone Soy Agar containing 5% sheep blood (Meus, Vacutest Kima s.r.l., Arzergrande, Padova, Italy) and the test isolate was streaked down perpendicular toward the center of the *S. aureus* streak [22,46]. To avoid contamination between *S. lugdunensis* isolates, these were tested in separate plates. The plates were incubated for 24 h at 37 °C and synergistic hemolysis reactions were detected. The test was performed in duplicate. A clear and large hemolysis zone bordering the *S. lugdunensis* strain under examination, within the zone of incomplete hemolysis near the streak of *S. aureus*, was positive evidence. On the other side, the *S. lugdunensis* that were negative for the δ-hemolysin produced a zone of incomplete hemolysis, which appeared homologous with hemolysis caused by the beta-lysin of the *S. aureus.*

## 5. Conclusions

The most significant outcome was the finding of an altered size of the *slush* operon gene in two isolates belonging to the same *cra*129-S8 ribogroup and to the cluster C as determined by BioNumerics analysis. This evidence can be useful to predict the genetic lineage of *S. lugdunensis* strains. The genetic lineages of the *S. lugdunensis* strains did not correlate with the gene expression of virulence traits except for the *hemolysin III* gene. The hemolytic and synergistic hemolytic activity phenotypes do not correlate with the presence of the *slush* operon gene nor with the gene expression of either *slush A*, *B* or *C* genes nor of *hemolysin III* or *β-hemolysin*. Consequently, it is desirable to continue searching for other possible pathological mechanisms.

## Figures and Tables

**Table 1:** Relation of the 24–72 h hemolysis, *S. aureus*/*S. lugdunensis* isolates synergy, and the logarithmic values of *slush* gene expression on the 22 *S. lugdunensis* isolates studied.

Strain	Ribogroup	24 h Hemolysis	48 h Hemolysis	72 h Hemolysis	*S. aureus* Synergy	Log *SlushA*	Log *SlushB*	Log *SlushC*
N940084	*cra*-62-S1	positive			positive	2.757	2.867	0.587
N940113	*cra*-62-S1	positive			positive	3.161	3.522	0.955
*cra*2847	*cra*-62-S1	negative	positive		positive	2.728	2.916	0.842
N940025	*cra*-62-S1	positive			positive	2.535	2.949	0.763
N940135	*cra*-62-S1	negative	positive		positive	2.696	3.095	0.890
*cra*1871	*cra*-62-S1	positive			positive	3.167	3.298	0.790
*cra*1894	*cra*-62-S1	positive			positive	2.691	2.673	0.850
*cra*1363	*cra*-62-S1	positive			positive	3.574	3.558	1.091
*cra*2653	*cra*-62-S1	negative	positive		positive	3.747	3.435	1.184
N920143	*cra*-62-S1	negative	negative	weak	negative	2.328	2.666	1.479
E319	*cra*-62-S1	negative	negative	weak	negative	0	0	0
N930432	*cra*-193-S3	negative	negative	weak	negative	2.154	3.219	1.009
N940164	*cra*-74-S5	negative	negative	weak	negative	0.403	3.047	1.080
*cra*1750	*cra*-74-S5	positive			positive	0	3.401	0.923
*cra*2501–1	*cra*-64-S8	positive			positive	3.325	3.220	0.779
*cra*2522	*cra*-64-S8	negative	positive		positive	2.667	2.707	0.891
*cra*2363	*cra*-64-S8	negative	positive		positive	3.207	3.347	0.875
*cra*2050	*cra*-64-S8	negative	positive		positive	2.848	3.132	0.737
*cra*2773	*cra*-64-S8	negative	positive		negative	3.688	3.979	0.929
*cra*3006	*cra*-64-S8	negative	negative	weak	positive	2.250	1.970	0
*cra*1342	*cra*-129-S8	positive			Positive	0	1.027	0.903
N860314	*cra*-129-S8	negative	positive		Positive	0	0	0

**Table 2:** Primers and probes used designed for qPCR of *slush* genes.

Primer/Probe Sequence (5′–3′)	Target Name	Amplicon Size
CAG GTA TCG TAG ATG CAA TTT CAA	*slushA* fw	126 bp
AGT TAA AGA AAC CAG CGA TAA AGT C	*slushA* rev	
/56-FAM/AA GCA TTG C + T + G A + TG CA + A TC/3IABkFQ/	Probe *slushA*	
TGT CAG GTA TCA TTG AAG CAA TTA C	*slushB* fw	119 bp
GCC TGA AAT TGC GTC AAT GC	*slushB* rev	
/56-FAM/AA + TGG + G + C + A CT + AGCA/3IABkFQ/	Probe slushB	
ATG GAC GGA ATT TTT GAA GCA	*slushC* fw	129 bp
GTG GAA TAA TCC AAT AAT AAA GTC TAC AC	*slushC* rev	
/56-FAM/AG + A + C + A AAG + A+C + T GGG C/3IABkFQ/	Probe *slushC*	
TCT AAA CTT GGT GGC GCT AAA	*GMK* fw	
CGA TGG AAG CTG GAC ATG AT	*GMK* rev	
/56-FAM/AG TGC GTC C/ZEN/G GGA ATT TCT TCC TT/3IABkFQ/	Probe *GMK*	

## Data Availability

The data presented in this study are available upon request from the corresponding authors (S.R. and C.R.A.).
